# Addiction and the Brain: Development, Not Disease

**DOI:** 10.1007/s12152-016-9293-4

**Published:** 2017-01-11

**Authors:** Marc Lewis

**Affiliations:** University of Toronto, 27 King’s College Circle, Toronto, ON Canada

**Keywords:** Addiction, Disease model, Learning, Development, Neuroplasticity, Self-organization, Delay discounting

## Abstract

I review the brain disease model of addiction promoted by medical, scientific, and clinical authorities in the US and elsewhere. I then show that the disease model is flawed because brain changes in addiction are similar to those *generally* observed when recurrent, highly motivated goal seeking results in the development of deep habits, Pavlovian learning, and prefrontal disengagement. This analysis relies on concepts of self-organization, neuroplasticity, personality development, and delay discounting. It also highlights neural and behavioral parallels between substance addictions, behavioral addictions, normative compulsive behaviors, and falling in love. I note that the short duration of addictive rewards leads to negative emotions that accelerate the learning cycle, but cortical reconfiguration in recovery should also inform our understanding of addiction. I end by showing that the ethos of the disease model makes it difficult to reconcile with a developmental-learning orientation.

The harm done by addicts to themselves and those around them has riveted public attention in recent years. It has become essential to discard outdated perceptions of addiction and replace them with coherent models based on scientific principles. Toward this end, doctors, psychiatrists, medical researchers and treatment providers have come to define addiction as a brain disease. Specifically, addiction is characterized by changes in brain systems that mediate the experience and anticipation of reward, systems responsible for perception and memory, and higher-order executive systems underlying cognitive control. The disease model stipulates that these changes are caused by exposure to drugs of abuse, and they are difficult if not impossible to reverse.

By looking at changes in the function and structure of the nervous system, the disease model helps explain why it is so difficult to achieve abstinence through the exercise of willpower. It makes sense of individual differences in vulnerability to addiction, based on dispositional factors and environmental stressors. The disease model provides a knowledge base and research agenda for developing pharmaceuticals that can be useful for reducing craving and easing withdrawal symptoms. And it has countered the perception that addicts are morally deficient or self-indulgent, arguably reducing the stress and isolation they and their families experience.

Given these achievements, it isn’t surprising that the disease model of addiction is accepted—in fact nearly unchallenged—by the medical community, the psychiatric community, research funding bodies, and governments themselves, as reflected by a mountain of articles and posts by the National Institute on Drug Abuse (NIDA), the National Institutes of Health (NIH), the American Medical Association (AMA), and the American Society of Addiction Medicine (ASAM). Yet there are reasons to question the validity of the disease perspective. First, this perspective clashes with the experience of many former addicts, who do not feel they were ever sick or have now been cured. Second, the strongest endorsements of the disease model come from the rehab industry and Big Pharma, both of which profit from the belief that addicts need long-term medical treatment. Rather, most alcoholics and addicts recover [1], and most of those do so without treatment of any kind [2–4], a finding that is difficult to reconcile with the idea that addiction is a chronic disease. Finally, investigators who approach addiction as a disease are far more likely to get their work funded, thus minimizing the volume and impact of discrepant findings.

For these and other reasons, the disease model of addiction has been heatedly challenged, and alternative models have been proposed in its place. Addiction may be viewed as a choice rather than a pathology. While few people imagine that addiction is a good choice, it is sometimes considered rational in the short run—as when the pleasure or relief derived from drugs temporarily outweighs the alternatives [5, 6]. Addiction may be a natural response to environmental or economic conditions beyond the addict’s control, including poverty and social alienation [6, 7]. Addiction can be viewed as a form of self-medication that works against psychological suffering. Trauma—whether physical, psychological, or sexual—is often considered the root cause of long-term anxiety and depression; and post-traumatic stress disorder (PTSD) is highly correlated with substance use [8–10]. A framework that encompasses all these approaches views addiction as a product of cognitive and emotional development, predisposed by constitutional factors but consolidated through learning over childhood and adolescence [10].

These alternatives to the disease model of addiction may be compelling, but they lack one important ingredient. They have little or nothing to say about the brain. (There are notable exceptions [11–13], which, although valuable, provide only global neural arguments, without attention to key structures or processes. Maia Szalavitz [10] is the only author I’m aware of who backs a learning account of addiction with detailed neuroscientific explanation.) In this era of scientific acceleration, brain science has become a gold standard for conclusive explanations of human phenomena. Without detailed neurobiological analysis, alternatives to the disease model may lack the scientific traction they need. My book, *The Biology of Desire* [14]*,* was intended to fill in the neural level of analysis in a developmental-learning model of addiction, integrate that level of explanation with experiential accounts of addiction and recovery, and demonstrate that the disease model has outlived both its credibility and its usefulness. In the following sections, I summarize these arguments and connect them to the larger debate on how to understand and combat addiction. I end by showing that the ethos of the disease model makes it difficult to reconcile with a developmental-learning orientation.

## The Core Tenets of the Disease Model

According to NIDA, “Addiction is defined as a chronic, relapsing brain disease that is characterized by compulsive drug seeking and use, despite harmful consequences.” A key observation underlying this depiction is that dopamine transmission and reception are altered over time: increasingly, it is only the user’s substance of choice that reliably impacts on dopaminergic activity. Dopamine is a crucial neurotransmitter (or “neuromodulator”) for motivating, directing, and rewarding goal-directed behavior and focusing attention and memory. Because the action of dopamine enhances the formation of new synapses (and the corresponding loss of older ones), changes in dopamine metabolism bring about structural changes in synaptic networks—the basic wiring diagram of the brain. A critical locus of dopamine reception and synaptic restructuring is the striatum, the area responsible for pursuing rewards, but other targets include the amygdala, which mediates emotional salience, the hippocampus, which directs memory encoding and retrieval, and several regions of the prefrontal cortex, responsible for a variety of cognitive functions.

Indeed, starting in the 1980s and 1990s, researchers began to show synaptic changes in these regions in laboratory animals exposed to cocaine, amphetamine, morphine, alcohol, and other drugs, corresponding with behavioral sensitization in addicted animals and humans [15, 16]. For example, dopamine activation of the striatum was found to go up and down with drug availability—and not much else. The receptors that absorb and use dopamine were also found to change in structure or efficiency [17] increasingly over months and years of use. The message seemed clear: drug use messes up brain wiring. These brain changes were seen as direct evidence that an insidious force—namely drugs—had “hijacked the brain,” a phrase first uttered by Bill Moyers on a popular PBS television series, but quick to catch on in addiction debates everywhere.

Nora Volkow M.D., the firebrand scientist who currently heads NIDA, points to “tissue damage” in the brain as indisputable support for the disease model [18]. In her view, this damage is specifically caused by drug use, and it corresponds with reduced capacity to engage cognitive control, increased compulsivity in drug seeking, and emotional blunting in response to rewards more generally. The *nucleus accumbens* describes one of the most ventral (lower) regions of the striatum, and it is the brain part most often referred to when it comes to addiction. Berridge and Robinson [19] coined the phrase *incentive sensitization* to describe the increasing specificity with which dopamine flows from the ventral tegmental area (VTA) in the midbrain to the accumbens in response to drug cues. In fact, even secondary and tertiary drug-related cues were found to trigger dopamine release, which then increased activation in the accumbens and induced a more driven, even “frenzied” quality to drug-seeking behavior [20, 21].

The ventral striatum or accumbens is associated with impulsive drug seeking and use, but the dorsal striatum becomes increasingly important for addiction with the passage of time. As the period of addiction stretches over months and years, activation shifts from the ventral to the dorsal striatum in response to drug-associated cues, while drug-seeking behavior becomes more compulsive and less impulsive in character. Trevor Robbins and his colleagues at Cambridge have been studying the shift from impulsive to compulsive drug seeking for many years [22]. They see the compulsive phase as true addiction, as do many others in the field. Now, according to Volkow, Koob, and others, the addictive urge is truly out of control. Whether the addict actually desires the addictive reward, he or she is compelled to go after it, based on a stimulus-response (S-R) association acquired and strengthened through Pavlovian conditioning. The stimulus simply elicits a response, without the need for a reinforcing outcome.

According to Volkow and other scientists, not only the brain regions underlying goal-seeking but also those responsible for self-control are physically modified by drugs. An example can be seen in the dorsolateral prefrontal cortex (dlPFC), which is critical for reasoning, remembering, planning, and self-control. The dlPFC becomes hyperactivated in the early stages of addiction, as it does in some eating disorders, perhaps when people try to control or maintain the rewardingness of this new experience. But over time, this region and other prefrontal control centers start to disengage (i.e., lose functional connectivity) from the striatum, the amygdala, and other areas comprising the motivational core of the brain [23, 24]. Volkow and colleagues have carried out two decades of research into cortical changes underlying addiction. They conclude that prefrontal regions responsible for judging options and selecting among them lose grey matter volume (reduced synaptic density) and become partially dysfunctional over the course of addiction [23, 25]. They dub the resulting cognitive dysfunction “impaired response inhibition.”

This cluster of changes in the function and structure of the brain has led many authorities to view addiction as a disease, and because these changes seem to endure long beyond the cessation of drug-taking, it is considered a chronic disease. According to Steven Hyman, previous director of the National Institute of Mental Health, addiction is a condition that changes the way the brain works, just like diabetes changes the way the pancreas works. Then why shouldn’t it be viewed as a disease?

## Development and the Brain

One of the key premises of the disease model is that addiction changes the brain. Yet brains are supposed to change. They are designed to change. In fact the stages of child and adolescent development, and the learning that goes on throughout adulthood, are all underpinned by changes in the cortex and limbic regions. Given the realities of brain change in normal development and learning, neuroscientists who endorse the disease model must view the brain changes resulting from addiction as extreme or pathological. In fact, they would have to show that the *kind* (or extent or location) of brain change characteristic of addiction is nothing like what we see in normal learning and development. How then should we characterize brain changes that occur naturally?

First of all, brains grow and shape themselves, not by following prespecified guidelines, but by a process of *self-organization*. They organize themselves, changing their own structure as they go. Such changes build on themselves over time, such that the products (synaptic changes) of one learning episode set the conditions for subsequent learning episodes. Of course there are some species-specific constraints on the timing of neural development, and there are certainly constraints on the kinds of information human beings can access and manipulate. Moreover, social norms help guide neural development along pathways consistent with particular cultural environments. Yet neural development is in no way programmed. It results almost entirely from synaptic activation patterns that both result from and give rise to experience itself.

One way to conceptualize this kind of self-perpetuating growth is to see it as a feedback loop between experience and brain change. The way we experience things changes synaptic configurations, and those changes shape the way we experience things subsequently. In other words, experience-dependent changes in brain structure make a particular way of experiencing things more probable on future occasions [26]. This can take the form of a self-perpetuating perception (as in language learning), an expectancy, a budding interpretation (as in judgments of individuals or groups), a recurring wish, a familiar emotional reaction (as in anxiety regarding perceived threats), an emergent belief (as in religious ideas and corresponding *ism*s), or a conscious memory. Thus the mind and the brain shape each other. And ordinary classroom learning is just one version of this more general phenomenon—a brain that changes itself (a phrase borrowed from Norman Doidge [27]).

The brain would be useless if it wasn’t highly changeable and highly sensitive to events in the world. But since we need stability in our percepts, concepts, and actions, brain changes almost always settle into habits. And once formed, habits—even minor habits—remain in place, sometimes for the rest of our lives. Examples range from idiosyncratic patterns like nail-biting and suspiciousness to cultural norms like politeness and sexual stereotyping. New synaptic pathways, and corresponding patterns of thought and behavior, start off tentative and fluctuating. But after they’ve been activated repeatedly, fledgling pathways get more entrenched, more concretized. As Donald Hebb made famous in the 1940s, *cells that fire together wire together.* Change and stabilization—novelty and habit formation—work together in the mind and in the brain. In a word, that’s “learning”.

Another helpful concept is *neuroplasticity.* Neuroplasticity simply describes brain changeability and elevates it to a first principle. Indeed, there’s nothing more fundamental to the human brain than its plasticity [27]. Yet neuroscientists who study addiction seem to have missed the point. When the brains of addicts (following years of drug taking) are compared to those of drug-naive controls, these scientists can be heard to say “Look! Their brains have changed!” Yet if neuroplasticity is the rule, not the exception, then they’re actually not saying much at all. The brain is supposed to change with new experiences. And those changes are supposed to stabilize and consolidate the more that experience is repeated.

When our experience of the world produces strong emotions—whether of desire, threat, pleasure, or relief—brain change takes on extra momentum. Emotions focus our attention and our thinking, partly through connections between the amygdala and a variety of cortical structures and partly through the wash of neuromodulators (including dopamine) released from the brain stem (including the VTA) in response to salient inputs. When those emotions recur over and over, in response to a particular event, perception, thought, memory, or need, then attention directs memory consolidation systematically. Our recurrently-focused brains inevitably self-organize in a particular direction, entrenching particular interpretations and emotional associations. Most relevant to addiction, the feeling of *desire* for something shapes synaptic configurations that become increasingly sensitive to cues associated with whatever is desired—since those cues are processed repeatedly in our efforts to acquire it.

Importantly, it’s not just attraction or desire that fuels feedback loops and promotes neural habits. Depression and anxiety also develop through feedback. The more we think sad or fearful thoughts, the more synapses get strung together to generate scenarios of loneliness or danger, and the more likely we are to practice strategies—often unconsciously—for dealing with those scenarios. Neural patterns forged by desire can complement and merge with those born of depression or anxiety. In fact, that’s a lynchpin in the self-medication model of addiction. Gabor Maté persuasively shows how early emotional disturbances steer us toward an intense desire for the relief provided by drugs [11], and Maia Szalavitz vividly portrays her experience as a late adolescent trying to brighten her depression with cocaine and ease her anxiety with heroin [10]. So, when we examine the correlation between addiction and depression or anxiety, we should recognize that addiction is often a partner or even an extension of a developmental pattern already set in motion, not simply a newcomer who happened to show up one day.

Thus, repeated experiences establish patterns, forming habits, and those habits link with other habits that also evolve with repeated experiences. But here’s the main point when it comes to addiction. We don’t need an external cause like *disease* to explain the growth of bad habits, or even a set of interlocking bad habits (like being a drug addict and a criminal and a liar). Bad habits self-organize like any other habits. Addiction has been described as a habit for many decades, across various cultural contexts and societal conversations. Is that all it is? Like other habits, addiction may simply grow and stabilize, in brain tissue that is designed (by evolution) to change and stabilize. Yet addiction belongs to a subset of habits: those which are most difficult to extinguish. If we conceptualize addiction as an outcome of normal learning, we still have to explain why it is such an extreme outcome, so destructive and so difficult to reverse.

My outline of the principles of brain development highlighted individual trajectories. However, brain development also incorporates normative tendencies that are crucial for understanding addiction. First, brain development always balances the formation of new synapses—synaptogenesis—with synaptic loss or pruning. Second, and perhaps counterintuitively, synaptic pruning far outweighs synaptogenesis over the years of childhood and adolescence. The infant brain has an overabundance of synapses, roughly one-third of which are pruned through competition [28] as a result of normal learning. In fact pruning is considered the primary mechanism by which learning occurs. Third, pruning in the prefrontal cortex increases efficiency in the processing and organizing of information—the essence of cognitive development from puberty onward [29]. Fourth, emotion regulation skills, which continue to advance through childhood and adolescence, involve two-way communication between prefrontal control centers and subcortical (e.g., striatal) regions that mediate emotions and impulses [30]. It can be assumed that both synaptogenesis and pruning play significant roles in this crucial developmental achievement.

A closer look at the nature of impulsive responding will help us understand not only the development of emotion regulation but addiction as well. All mammals and certainly human children tend to overvalue immediate rewards at the expense of long-term gains. This proclivity, called delay discounting, must be tamed in order for children to advance from a preoccupation with whatever is presently available (e.g., one marshmallow in the famous marshmallow test) to a capacity to wait for long-term gains (e.g., two marshmallows, a few minutes later) [31]—a crucial step in the development of emotion regulation. Addicts are known to be excessively now-oriented [32], consistent with their tendency to favor what Heyman calls the local choice [5]. Moreover, delay discounting has been shown to correspond to activation of the ventral striatum, the villain when it comes to addictive behavior, while the capacity to delay gratification taps activation of the dlPFC [30, 33, 34]. In other words, the neural picture in both delay discounting and addiction features striatal activation that is underregulated by the dlPFC (and other regions of the PFC).

## Why Addiction Is Not a Disease

In its contemporary form [18], the disease model of addiction asserts that addiction is a chronic, relapsing brain disease. This disease is evidenced by changes in the brain, especially alterations in the striatum, brought about by the repeated uptake of dopamine in response to drugs and other substances. But it is also characterized by changes in the prefrontal cortex, where regions responsible for cognitive control become partially disconnected from the striatum and sometimes lose a portion of their synapses as the addiction progresses. These are big changes, they can’t be brushed aside, and so far the disease model is the only model of addiction that actually tries to explain them. So why should we look further?

### Self-Perpetuating Attractions Do Not a Disease Make

The brain changes with all learning experiences, and it changes more rapidly and more radically in response to experiences with high motivational impact. Every experience that is repeated enough times because of its motivational appeal will change synaptic networks in the striatum and related regions (e.g., the amygdala and orbitofrontal cortex) while adjusting the flow and uptake of dopamine to all these regions. Such changes lead to the formation of habits—neural and behavioral habits—habits that become self-perpetuating and self-stabilizing. Yet we wouldn’t want to call the excitement we feel about summer vacation, meeting our lover, or cheering for our favorite team a disease. As we anticipate and live through these experiences, the corresponding network of synapses is strengthened and refined; so the uptake of dopamine gets more selective as rewards are identified and habits established. This is simply learning, motivated by desire.

Even if addictive habits are more deeply entrenched than other habits, there is no clear dividing line between addiction and the repeated pursuit of other attractive goals, either in experience or in brain function [35]. So how do we know which urges, attractions, and desires to label “disease” and which to consider aspects of normal experience and brain change? Some authorities apply the disease label when the pursuit of a drug, drink, or activity seriously interferes with one’s life. But again, where should we draw the line? The lover we can’t help but desire may be abusive, may be involved in another relationship, or may be forbidden for familial or cultural reasons. And sports fans have been known to beat each other up, get arrested, and ignore their familial responsibilities when the excitement runs high. “Addiction” doesn’t fit a unique physiological stamp. It simply describes the repeated pursuit of highly attractive goals and the brain changes that condense this cycle of thought and behavior into a well-learned habit. Brain change, even more extreme brain change, does not imply that something is wrong with the brain.

My review of the disease model highlighted the shift in activation from the ventral to the dorsal striatum as addictive behavior becomes increasingly compulsive. This change has been well documented: it consists of the growth of fibers from the VTA to the dorsal striatum as the addictive behavior becomes an automatic response to a stimulus [22]. Once a person has reached this state, the brain is no longer functioning as it did. Yet, according to Everitt and Robbins [22], the acknowledged experts on the ventral-to-dorsal shift, “there is nothing aberrant or unusual about devolving behavioural control to a dorsal striatal S-R habit mechanism.” These authors remind us that this neural restructuring is to be expected in many aspects of our lives, including eating and other normal activities. Do we bite down on that piece of pizza because of an anticipated reward, or because a great many trials have established an association between a particular smell (and other gustatory cues) and the act of biting? “Automatization of behaviour frees up cognitive processes,” these authors continue. That would explain why we can talk, eat, drive, and listen to music all at the same time. We need habits in order to free our minds for other things. Unfortunately, in addiction, this perfectly natural developmental mechanism often leads to suffering.

### Addiction without Substances

One of the greatest blows to the current notion of addiction as a disease is the fact that behavioral addictions can be just as severe as substance addictions. However, the party line of NIDA, the AMA, and ASAM remains what it has been for decades: addiction is primarily caused by substance abuse. If that were so, how would we explain addictions to porn, sex, internet games, food, and gambling? In a comprehensive review, Brewer and Potenza conclude that “disorders” characterized by too much of any of the above show brain activation patterns that are nearly identical to those shown in drug addiction [36]. According to these authors, even the ventral-to-dorsal shift in striatal activation, and the corresponding increase in compulsive responding, show up in behavioral addictions just as they do in substance addictions. This is exemplified in compulsive gambling and binge eating. It is interesting that, despite widespread acceptance of neural and behavioral parallels between substance and behavioral addictions, the promoters of the disease model have never retracted their claim that drugs cause the brain changes underlying addiction.

People pursue certain activities repeatedly, often with little control, because those activities start off as highly rewarding and end up as behavioral habits. That description can cover anything from spending sprees to helicopter parenting to jihadism. But there is one very normal human endeavor that most of us recognize as the epitome of blind desire and recurrent pursuit: falling in love. Lovers think obsessively about their love object, exaggerate his or her positive qualities and avoid thinking about future repercussions. Romantic love (but also parent-child love, and even perverse forms of love including fetishism, sadomasochism, etc.) can easily become compulsive, difficult to control, and overly focused on the immediate, with little regard for the long-range forecast.

A look at the neuroscience of love reveals some remarkable similarities with addiction. It is generally agreed that “increased levels of central dopamine contribute to the lover’s focused attention on the beloved and the lover’s tendency to regard the beloved as unique” [37]. In fact, several researchers have examined the love-and-addiction link directly. Burkett and Young reviewed much of this work [38]. In their words, “mesolimbic dopamine is a major contributor to the formation of pair bonds in prairie voles and particularly in the nucleus accumbens region.” In a comprehensive new book, Toates summarizes research showing that the dopamine system provides a “common currency of wanting” in the pursuit of financial gains, drugs, and sexual partners [39]. He notes that the nucleus accumbens is involved in motivating the individual to overcome obstacles in order to reach such goals [40] and that dopamine metabolism biases decision making in favor of immediate gains [41]. With regard to romantic pairing, Burkett and Young conclude that “[w]hen these early interactions with the object of addiction produce rewarding outcomes, dopamine is released in the nucleus accumbens, which acts to increase the salience of incentive cues that predict the reward” [38]. If addiction is a disease, then so apparently is love.

### Alternative Explanations of Cortical Change

So far, I’ve argued that addictions are consolidated patterns of attraction and pursuit that cultivate distinct synaptic configurations in the motivational core of the brain (the striatum and related regions). But the disease model also stipulates cortical changes: most seriously the loss of functional coupling between the PFC and the striatum and, perhaps as a result, the eventual loss of synapses in the PFC, both of which contribute to a loss of self-control. Indeed, after a while, with a variety of substances and some eating disorders (including binge eating), the dorsolateral PFC becomes partially disconnected from the striatum. The reasons for this disconnection are complex and not fully understood. But suffice it to say that dopamine signaling in the cortex is partly under the control of striatal outputs, and with long-term addiction striatal habits no longer send signals to the PFC eliciting control. Functional connections are lost, which means some of the synaptic pathways get pruned and eventually disappear. Now structural connections are lost. This explains the loss of grey matter volume reported with long-term addiction. Can these changes be seen as anything but the ravages of a disease?

From a functional perspective, the interplay between prefrontally mediated control and striatal goal-pursuit is never permanently fixed in the brain. Children’s ability to overcome delay discounting (and other impulsive tendencies) improves with age from middle childhood to middle adolescence, due at least in part to the maturation of the dorsolateral PFC [42]. Not surprisingly, adults also overcome delay discounting by activating the dlPFC [33], yet this avenue of control isn’t carved in stone. Adults fall prey to delay discounting regularly, suggesting functional rather than structural variability in prefrontal control. And they can reverse this tendency in response to novel environmental inputs. In one set of studies, the tendency to discount future gains in favour of immediate rewards was consistently reversed by exposing participants to images of their future selves [43]. To examine such changes at the neural level, Figner applied transcranial magnetic stimulation (TMS), a procedure that can temporarily disrupt activity in the cortex, while participants were engaged in a delay discounting task [44]. Participants chose immediate rewards of lower value more frequently when the TMS machine was placed over their dorsolateral PFC, but their discounting rate went back to normal immediately afterward. There are more natural (and less expensive) ways to disrupt dlPFC activation and facilitate impulsive responding. Drug or alcohol use, especially during the sensitive developmental period of adolescence, is clearly one such way [45].

Yet the loss of cortical control is thought to be long-lasting, even permanent, in long-term addiction. This implies structural changes, which are often conflated with the notion of disease. However, as noted previously, synaptic pruning is a normal developmental process. In fact, research shows that, when the same inputs are encountered repeatedly, connections are depleted to improve overall efficiency [46], and addiction certainly exemplifies repeated inputs. In the sequel to Hebb’s famous maxim, not only do cells that fire together wire together but cells that fire apart wire apart. In other words, changes in behavior and experience naturally deplete synaptic connections, not only functionally but, over time, structurally as well. As addicts pursue the same rewards every day, it appears that they no longer rely on reflective judgment to curtail the feelings and behaviors to which they’ve grown accustomed. Then it should not be surprising, nor should it imply the presence of disease, if their neural configurations readjust by pruning the underused synapses.

This account of cortical decoupling and loss of cortical synapses doesn’t quite close Pandora’s Box. It isn’t easy to determine which patterns of synaptic pruning are normal and which are not [47]. Yet, in a seminal study, Connolly and colleagues showed that the reduction of grey matter volume in specific regions of the prefrontal cortex (and the anterior cingulate, a closely related structure), induced by years of addiction, can reverse over several months of abstinence [48]. These authors reported that grey matter volume returned to a normal (population) baseline level within six months to a year of abstinence (from heroin, cocaine, and alcohol), and similar results have been found by others [e.g. 49]. Of even greater interest, Connolly and colleagues observed an increase in grey matter volume *beyond* the population baseline in participants who remained abstinent for a year or more. These findings jibe with the idea that synaptic loss and synaptic growth in these regions correspond with variations in experience, not disease. Recurrent episodes of automatic responding reduce synaptic activity in the PFC, but new modes of experiencing the world and new means for regulating one’s emotions and behaviors can just as easily build new synaptic connections in the same (or nearby) regions.

From subjective reports we know that most addicts never feel that they have lost all control over their impulses. Rather, most addicts report that control has become more difficult because it is buffeted by a variety of psychological and social factors: it has become less automatic—more nuanced but less reliable [50]. And from epidemiological reports the story is clear: most addicts recover [1], and most of those recover without treatment [2–4]. This would seem impossible if regions of the PFC responsible for self-control did not remain highly plastic.

In fact, a detailed understanding of neuroplasticity is the best antidote to the disease model of addiction. Yes, the prefrontal cortex is malleable. Yes, it can undergo major changes in synaptic organization in response to drug taking. But it can and must undergo synaptic reorganization anyway, and it does so throughout a lifetime of learning. Spontaneous recovery from addiction is common, it has been studied in depth, and it certainly must embody cortical plasticity, though in a direction opposite to that highlighted by disease model advocates. Neuroplasticity (e.g., synaptogenesis) is the norm when people recover from medical problems like strokes or concussions [27, 51], but it also underpins second language learning [52] and the acquisition of new skills in adulthood. People *learn* addiction through neuroplasticity, which is how they learn everything. They maintain their addiction because they lose some of that plasticity. Then, when they recover, with or without treatment, their neuroplasticity returns. Their brains start changing again. With the onset of addiction, plasticity is devoted to new means for acquiring pleasure or relief. With recovery, plasticity is devoted to goals with far-reaching personal value and the skills necessary to attain them.

## If it’s Not a Disease, then What Is it?

In an earlier section, I outlined a number of processes by which brains change as people (and their habits, and their personalities) develop. The repetition of particular experiences modifies synaptic networks. This creates a feedback cycle between experience and brain change, each one shaping the other. New patterns of synaptic connections perpetuate themselves like the ruts carved by rainwater in the garden. Thus, brain changes that result from repeated learning experiences naturally settle into brain habits—which lock in mental habits. And the experiences that get repeated most often, most reliably, are those that are most compelling. In fact, *desire* is evolution’s premier agent for getting us to pursue goals repeatedly. Thus, intense and/or recurrent desires will naturally change the *rate* and *depth* of learning by augmenting the feedback cycle between experience and brain change.

In this sense, I would say that addiction is an outcome of learning, but learning that has been accelerated and/or entrenched through the recurrent pursuit of highly attractive goals. There are many reasons why this cycle of goal pursuit, accompanied by the fadeout of alternative goals, becomes tighter and more invariant over time. Some are social and cultural, others societal and economical. The reasons I have highlighted in this article have more to do with the cascading nature of developmental constraints—the narrowing of possibilities into probabilities, states into traits [53]. Looked at from a biological perspective, this tendency is embodied in the reconfiguration, self-perpetuation, and consolidation of synaptic networks in structures that mediate desire, attraction, attention, memory, and cognitive reflection and control [54, 55].

Desire is at the top of the list when it comes to emotional states that propel learning. And while this standard feature of the psychological repertoire can explain the locking in of habitual attractions, we must still ask whether there is something special about addiction that makes it so difficult to overcome. In fact, there seem to be at least three specific mechanisms that accelerate our attraction to addictive rewards and entrench addictive activities—without making it a disease.

The first is the tendency toward delay discounting, which creates a narrowed beam of attention toward imminent rewards. That is precisely the state addicts find themselves in time after time. One of dopamine’s chief functions is to highlight available goals. Immediate goals are available goals, and striatal networks surge with dopamine whenever those goals are cued by associated stimuli or memories. Another function of striatal dopamine is to inhibit awareness of competing goals (e.g., going out on a date, finding a movie to watch). In fact, that’s how the striatum narrows the beam of attention. As a result, addicts become stuck in a bleak here-and-now, nearly identical from one day to the next. It is this entrapment in the immediate that calls for treatment approaches that might help addicts stretch their sense of personal time, consistent with Ainslie’s powerful concept of *intertemporal cooperation* [56]*.* Movement in this direction can be facilitated by some form of interpersonal scaffolding (e.g., targeted dialogue in group or individual therapy) intended to hold this cooperation in place—until the addict can recreate it at will.

The second mechanism is the motivational *amplification* caused by addictive rewards. We know that synaptic patterns get reinforced with each repetition of the same kind of experience, whether it’s playing the piano, baking bread, or smoking crack. And we know that repetition boosted by strong motivation is the most effective driver of synaptic shaping. (Actually, strong motivation determines not only the frequency of repetition across occasions but also the resilience or purity of attention within occasions.) Then imagine the impact of a longed-for reward that only lasts a few hours, or maybe just a few minutes. Drugs wear off, drinking sedates, the money’s spent, or sexual pyrotechnics become boring. Addictive rewards whet the appetite and leave frustration, loss, and often depression in their wake. Moreover, because they are universally perceived as selfish and indulgent, they unleash great gouts of shame [50]. Because shame is such a painful emotion, it exacerbates the need for resolution, regulation, or escape.

In a nutshell, addictive rewards pack a double whammy. Desire flares again after only a few hours, a day at most, and brings with it a host of other compelling emotions. Physiological consequences, including withdrawal symptoms with certain drugs, make it a triple whammy. The cycle of acquisition and loss then recurs with increasing frequency, the same neural passages get dredged again and again, and the trajectory of learning is progressively reinforced.

The third mechanism that enhances addictive learning is the fusion between personality development and the consolidation of addictive habits. Not only desire but also negative emotions, like anxiety and shame, fuel synaptic configurations that strengthen themselves over development, as in the crystallization of depressive or anxious personality traits. The addictive habit thus converges with other habits consolidating within one’s personality, such that addiction complements or reinforces preexisting tendencies. Synaptic networks are not only self-reinforcing but also mutually reinforcing, in a brain that likes to conserve structure and resources, as do all living things. The mechanics of this process involve multiple brain regions, interlaced to form a web that holds the addiction in place—as part of one’s personality structure. Thus, intense emotions, focused attention, and cognitive habits harness one another, and together they gouge deep ruts in the neural underpinnings of the self.

So, what exactly is addiction? It’s a habit that grows and self-perpetuates relatively quickly, when we repeatedly pursue the same highly attractive goal. Or, in a phrase, *motivated repetition that gives rise to deep learning.* Addictive patterns grow more quickly and become more deeply entrenched than other, less compelling habits, because of the intensity of the attraction that motivates us to repeat them, especially when they leave us gasping for more. Often, emotional turmoil during childhood or adolescence initiates patterns of personality development that anchor the search for addictive rewards, serving as sources of relief and comfort. But there are other points of entry too, based on various intersections of dispositional and environmental factors. However it is entered, and however it is eventually left, addiction is a condition of recurrent desire for a single goal, but also an aspect or phase of personality development that leaves enduring footprints in neural tissue.

## Why can’t we just Get along?

Will a developmental-learning model of addiction ever make peace with the disease model? That would provide one kind of happy ending. It would encourage proponents of the disease model and those who study the development of addiction to talk with each other, share data and ideas, and derive higher-order explanations. Yet I don't think this is in the cards. Not because the disease model is so far off base scientifically. Some of the brain changes observed in addiction may be sufficiently ominous to exemplify both pathology and learning, as is the case in autism and schizophrenia. In fact, defining a category at the intersection of pathology and development is the stated goal of the burgeoning field of “developmental psychopathology” [57]. As with depression and anxiety disorders, the delineation between learning and pathology is not a line but a zone.

Yet the baggage accompanying the disease model may preclude a happy marriage. Society’s understanding of addiction can be seen as advancing through three broad stages (a somewhat similar model has recently been proposed [58]). First, beginning in the Victorian era, addicts were considered morally flawed and indulgent, sinners by choice or by happenstance. The appropriate response to addiction was to punish the addict through scorn, isolation, disenfranchisement, or incarceration. The proper resolution to the problem of addiction was to shame and punish the addict who might, with luck, go back to being good. This set of beliefs and attitudes was gradually overwritten by the disease model of addiction in the middle of the twentieth century. This change was driven by the emphasis on helplessness in Alcoholics Anonymous, beginning in the 30s, and the evolution of residential treatment centers that stressed obedience to therapeutic regimes, beginning in the 50s. Finally, the proliferation of neuroscience in the 80s and 90s sealed the deal by specifying the substrate of the disease, namely the brain. Now specific neural changes could be pinpointed as the source of addiction, and the disease model reached its zenith.

According to the disease model, the appropriate solution to addiction is to be found in the realm of medicine. Specifically, addicts should be urged (convinced or compelled) to follow the advice handed down by medical practitioners. As emphasized by Nora Volkow in dozens of policy statements, the solution to addiction isn’t shame. Rather than confess to being immoral, addicts are advised to confess to being incapable. The only hope to control addiction is to accept a regime imposed from outside, from the halls of medical authority, in order to subdue a problem located on the inside, in the mind itself (an approach to the treatment of mental disorders that has governed psychiatry throughout its history—with some unfortunate consequences). It is this baggage that seems destined to clash with the ethos of a third, more progressive view of addiction.

What I see as the third stage in our understanding of addiction is not restricted to reinterpreting the role of choice [58], though that’s part of the package. Rather, it’s a developmental model of the kind outlined in this article, highlighting a learning trajectory that consolidates in habitual patterns of thinking and feeling. This view of addiction admits the potency of social factors, like isolation and dislocation [59]. It makes sense of the impact of adversity in early development, as demonstrated by large epidemiological studies from the 80s to the present. It is consistent with a far more nuanced view of addiction, embodying personal, philosophical, and societal factors, as elaborated in a recent special issue of *Frontiers in Psychiatry* [60]. And finally, it builds on our advancing knowledge of the neurobiology of individual differences in development [57, 61].

According to a developmental-learning conceptualization, the appropriate response to addiction is neither shame and isolation nor submission to a therapeutic regime. Rather, it is further growth. The cure for addiction can’t be a medical regime that returns the addict to some previous level of stability or homeostasis. Rather, growth beyond addiction exemplifies developmental progress, powered by one’s own efforts. In this light, addiction can be viewed as a stage of individual development, and it must therefore be addressed through individual strivings based on individual perspectives, goals, and capacities. A developmental-learning model of addiction suggests that positive change must be conceived and pursued from within.

The final two stages in our understanding of addiction, the disease model and the developmental-learning model, achieve some of their plausibility on the basis of brain research. But the role of neuroscience in these two stages of conceptualization could not be more different. Neuroscience helped shore up the disease model by identifying deviations from what is considered standard neural architecture. Although it’s never been made clear exactly how this standard could be determined, we could say that the project of the brain disease model draws on the principle of “neuronormativity.” In contrast, the developmental-learning model embodies our advancing conception of *neuroplasticity.* A project focused on neuroplasticity replaces the search for norms with an emphasis on the brain’s capacity to change, and it confirms our intuition that there are many different ways to move forward [10, 14].

Thus, both models borrow something from neuroscience—a detailed breakdown of the biological landscape underlying addiction. But they are fundamentally different in their perception of that landscape. The brain is either a normative thing that can go wrong and then be repaired, or it is an open system that can develop in a multitude of directions, integrating the meaning of experience according to its own proclivities. No doubt this process of integration can be greatly facilitated by the cognitive scaffolding and emotional support provided by other people. Yet, neither the spirit nor the specifics of change can be dictated, either by professional authorities or by society in general. Since addiction is viewed as a phase of individual development, so is the pathway most of us find for moving beyond addiction.
